# Regulation of Apoptotic Pathways by *Stylophora pistillata* (Anthozoa, Pocilloporidae) to Survive Thermal Stress and Bleaching

**DOI:** 10.1371/journal.pone.0028665

**Published:** 2011-12-14

**Authors:** Hagit Kvitt, Hanna Rosenfeld, Keren Zandbank, Dan Tchernov

**Affiliations:** 1 Marine Biology Department, The Leon H. Charney School of Marine Sciences, University of Haifa, Mount Carmel, Haifa, Israel; 2 The Interuniversity Institute of Eilat, Eilat, Israel; 3 Israel Oceanographic and Limnological Research, National Center for Mariculture, Eilat, Israel; King Abdullah University of Science and Technology, Saudi Arabia

## Abstract

Elevated seawater temperatures are associated with coral bleaching events and related mortality. Nevertheless, some coral species are able to survive bleaching and recover. The apoptotic responses associated to this ability were studied over 3 years in the coral *Stylophora pistillata* from the Gulf of Eilat subjected to long term thermal stress. These include caspase activity and the expression profiles of the *S. pistillata* caspase and Bcl-2 genes (*StyCasp* and *StyBcl*-2-like) cloned in this study. In corals exposed to thermal stress (32 or 34°C), caspase activity and the expression levels of the *StyBcl*-2-like gene increased over time (6–48 h) and declined to basal levels within 72 h of thermal stress. Distinct transcript levels were obtained for the *StyCasp* gene, with stimulated expression from 6 to 48 h of 34°C thermal stress, coinciding with the onset of bleaching. Increased cell death was detected *in situ* only between 6 to 48 h of stress and was limited to the gastroderm. The bleached corals survived up to one month at 32°C, and recovered back symbionts when placed at 24°C. These results point to a two-stage response in corals that withstand thermal stress: (i) the onset of apoptosis, accompanied by rapid activation of anti-oxidant/anti-apoptotic mediators that block the progression of apoptosis to other cells and (ii) acclimatization of the coral to the chronic thermal stress alongside the completion of symbiosis breakdown. Accordingly, the coral's ability to rapidly curb apoptosis appears to be the most important trait affecting the coral's thermotolerance and survival.

## Introduction

At elevated seawater temperatures, scleractinian corals lose their endosymbiotic dinoflagellates (*Symbiodinium* spp; i.e., zooxanthellae), which leads to a bleached appearance (pale or white color) and often to death. Bleaching events appear to cause up to 60% mortality among a wide variety of tropical coral species and are deemed responsible for the extinction of almost 16% of coral reefs worldwide [1], [2]. However, some coral species are known to be able to survive and recover from bleaching [3]. Although this resistance has been attributed to coral morphology [4] or energy reserves and heterotrophic capability of the coral host [5], such resilience is in fact poorly understood and the role of the host remains unclear [6].

General mechanisms have been proposed to explain the thermal sensitivity of symbiotic cnidarians, resulting in bleaching [7]. One hypothesized mechanism of coral bleaching involves the increased production of reactive oxygen species (ROS) in the dinoflagellate symbionts, which would cause cellular damage and expulsion of symbionts [8]. ROS could diffuse into the host tissues, leading to oxidative stress [7], [9], [10]. As one of the signals for programmed cell death (PCD) [11], [12], ROS could be involved in the initiation phase of apoptosis, resulting in coral death.

PCD is a cell deletion mechanism that destroys redundant, dysfunctional, damaged, and diseased cells. This intrinsic process is of fundamental importance in the development, growth, health, and tissue homeostasis, and is highly conserved in all multicellular organisms. The form of PCD named apoptosis is characterized by activation of highly selective cysteine aspartate-specific proteases, known as ‘caspases’, that are constitutively expressed as proenzymes with low basal catalytic activity and are activated following appropriate stimulation. Caspases cleave a variety of cellular substrates, giving rise to several characteristic morphological features of apoptosis [12]–[15]. The cell death activation is governed by the protein-protein interactions of anti- and pro-apoptotic members of the B-cell lymphoma 2 (Bcl-2) protein family [16]–[18]. In metazoans, the Bcl-2 proteins act as a critical checkpoint for apoptotic cell death, regulating the permeability of the outer mitochondrial membrane [18] and also as regulators of oxidative stress [12]. Apoptosis has been remarkably well-conserved throughout metazoan phyla both in terms of morphological cell features and the repertoire of genes controlling the process [19]. This conservation appears to also include the most primordial metazoan phyla, such as Porifera and Cnidaria [20]–[25].

The correlation between thermal stress, oxidative stress and apoptosis (indicated by cell morphology, caspase activity and gene expression) has been demonstrated in symbiotic sea anemones [25], [26]. A correlation between thermal stress and the number of host cells exhibiting apoptosis was observed in corals [27]–[30]. In corals subjected to thermal stress, apoptosis (indicated by induction of caspase activity, DNA fragmentation and caspase protein levels) was negatively correlated with the species' ability to survive thermal stress and bleaching [31]. In addition, when the caspase cascade was interrupted in the host coral, the colony was “rescued” from apoptosis and only a moderate bleaching was observed, indicating the possible involvement of caspases and apoptosis in mechanisms that dictate the fate of the coral colony (i.e., death or recovery). However, no caspase genes, necessary for execution of host apoptosis, have yet been cloned from corals. Therefore, the objective of the current research was to study the apoptotic responses (i.e. caspase activity and apoptosis-related genes expression) in the stony coral *Stylophora pistillata* subjected to moderate and severe long term thermal stress, in which way they could be correlated to the ability of the coral to survive and recover from bleaching.

## Results

### Thermal tolerance and bleaching

**Figure 1:**
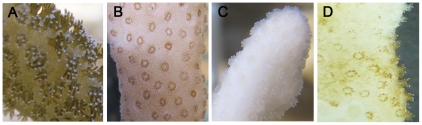
Morphology of coral polyps at different stages of thermal stress and recovery. Fragments of *S. pistillata* were placed in an aquarium and the temperature was raised to 34°C for 24 h and then back to 32°C for 1 month (Exp. 3), then ambient temperature (24°C) was restored. Pictures were taken at characteristic stages of thermal stress: (A) 0 h, (B) 24–48 h and (C) 168 h. (D) one month after ambient temperature was restored.

In order to test thermotolerance and set the bleaching threshold of *S. pistillata* from the Gulf of Eilat, corals were subjected to long term thermal stress and temperatures of 32°C, 34°C and 35°C. At 35°C the corals lost all recognizable tissue within 24 h and the experiment was aborted. Therefore, 34°C was defined as the upper thermal limit. In all other experiments, the corals survived up to one month at 32°C, then ambient temperature was restored; however, only when corals were exposed to 34°C bleaching was observed (Exp. 3). The coral polyps exposed to 34°C displayed a shrunken morphology while still harboring symbionts, but reverted to normal appearance once bleached (Fig. 1). Bleaching was observed after 72 h of stress and confirmed after 168 h by quantifying the number of zooxanthellae per cm^2^. In bleached corals, the number of zooxanthellae cell declined by 70% (Fig. S1).When placed at ambient temperature, the bleached corals slowly recovered, recovering back symbionts, as observed by change of color from white to green/brown (Fig. 1D). Recovery (i.e. gain of color similar to the control) was observed within 3 months.

### Detection of caspase activity in tissues of S. pistillata and TUNEL assay

**Figure 2:**
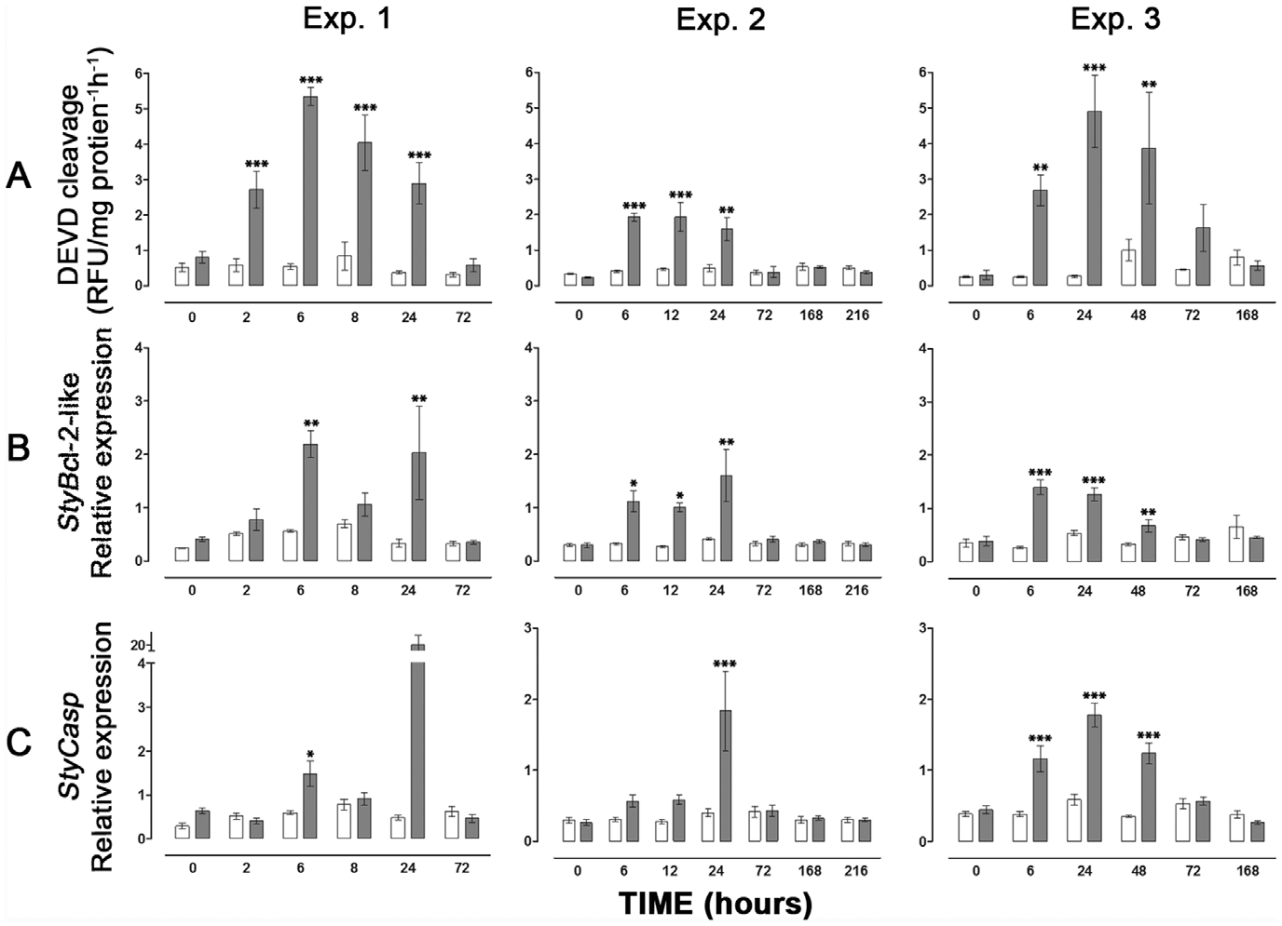
Caspase activities and quantitative analysis of *StyBcl*-2-like and *StyCasp*. Fragments of *S.pistillata* were placed in two aquaria (control at 24°C and thermal stress at 32°C in Exp. 1 and 2, or 34°C in Exp. 3). Fragments from both aquaria were sampled over 72 h (Exp. 1, n = 4), over 216 h (Exp. 2, n = 6) or over 168 h (Exp. 3, n = 6). Results are expressed as means ± SE of independent extractions from distinct fragments incubated in control (white) or subjected to thermal stress (gray). Values were tested by One-Way ANOVA and a t-test. Asterisks indicate significant difference between control and thermal stress of the same time point (* represents P<0.05, ** represents P<0.01, and *** represents P<0.001). (A) Caspase 3-like activities were assayed by fluorometric method using Ac-DEVD-AFC, measured in relative units of fluorescence (RFU's), and expressed as RFU/µg protein^−1^ h^−1^. (B), (C) Quantitative analysis of *StyBcl*-2-like and *StyCasp* expression, respectively, normalized to adenosyl-homocysteinase (AdoHcyase).

**Figure 3:**
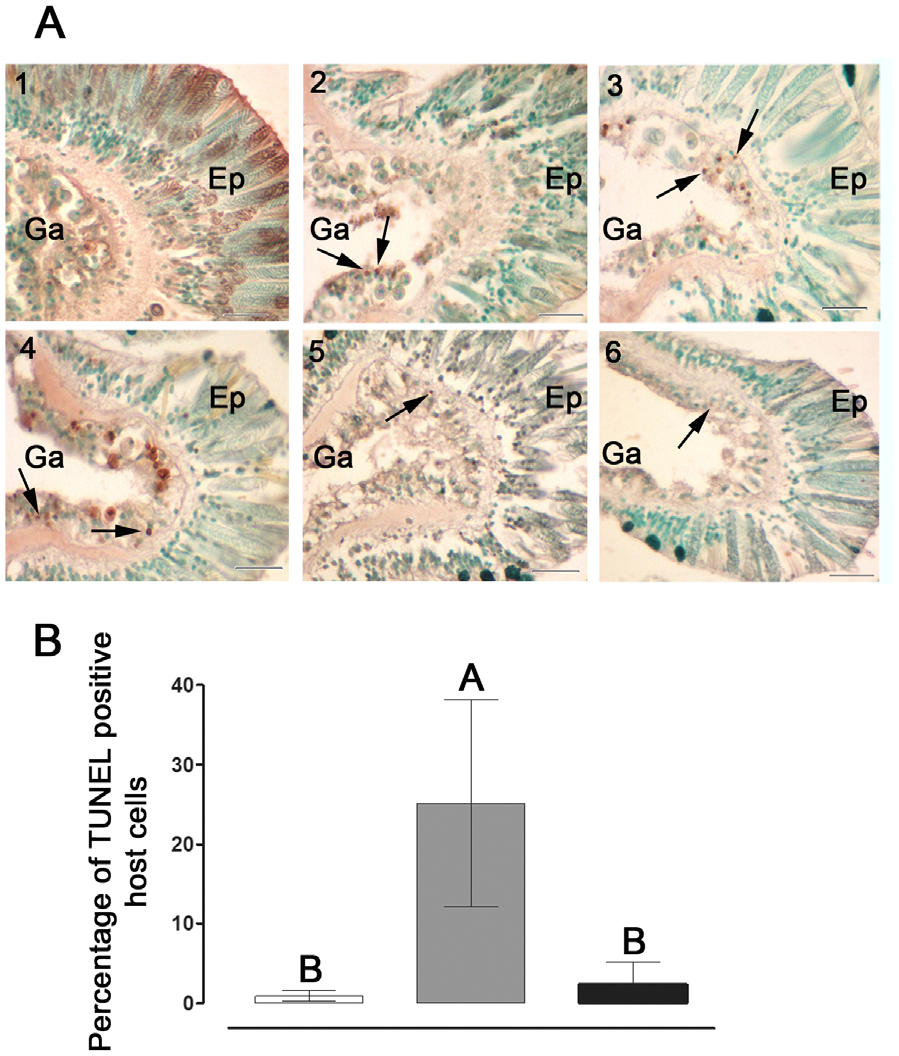
Detection and quantification of apoptosis-like activity in *S. pistillata*. (A) Temperature-induced DNA fragmentation detected *in situ* in tissues of *S. pistillata*. Fragments were incubated in control (24°C) or thermal stress (34°C). Sections (7 µm-thick) were attached to slides and apoptotic cells were detected using the TUNEL technique. (1) control; (2) 6 h, (3) 24 h, (4) 48 h, (5) 72 h and (6) 168 h of thermal stress. Arrowheads indicate apoptotic nuclei stained brown, while intact nuclei are counterstained green. Scale bar, 20 µm. Ep, epithelium. Ga, Gastroderm. (B) Percentage of TUNEL positive cells in tissues of *S. pistillata* incubated in control (24°C, white) or subjected to thermal stress of 34°C for 24 h (gray) and 168 h (black). Sections (7 µm-thick) were attached to slides and apoptotic cells were detected using the TUNEL technique. The mean percentage of TUNEL positive cells of individual fragments (n = 3) was derived by comparing the number of TUNEL positive cells to the total number of cells in the same image field. A total of five image fields per fragment section was analysed and averaged. Means were compared using a One-Way Anova with Tukey post-hoc testing. Letters above bars denote statistical significance; two means are significantly different (P<0.05) if their letters are different.

In order to test for the presence of caspase 3-like activity in *S. pistillata*, Z-DEVD-AFC (caspase 3 specific substrate) dependent protease activity was tested in host coral cells (Fig. 2A). In all experiments, caspase 3-like activity in the thermal-stressed groups (32 and 34°C) increased significantly over time, reaching maximal levels within 6–24 h of stress, i.e. 4- to 10-fold higher compared to controls. This activity declined to basal levels within 72 h of onset of thermal stress, remaining low up to 216 h. To confirm the induction of caspase-like activity, a universal and irreversible caspase inhibitor Z-VAD-FMK was added to selected samples. This addition totally abolished the caspase activity. Additionally, cell death was confirmed by the analysis of DNA fragmentation (TUNEL) assay in tissue layers of *S. pistillata* following exposure to 34°C (Fig. 3A). A relatively high number of TUNEL positive cells were observed following 6 to 48 h of thermal stress, as compared to the controls (Fig. 3A, 2–4). However, at 72 and 168 h of stress very few TUNEL positive cells were observed (Fig. 3A, 5–6). DNA fragmentation occurred largely in the gastrodermal tissue harboring the zooxanthellae. The percentage of TUNEL positive host cells detected at 24 h of stress (13 to 25%) was significantly higher compared to the controls (0.2 to 0.8%) and to 168 h stress (2 to 3%) which did not differ from each other (Fig. 3B).

### Cloning and sequence analyses of S. pistillata Bcl-2 family member (StyBcl-2-like) and caspase (StyCasp) genes

**Figure 4:**
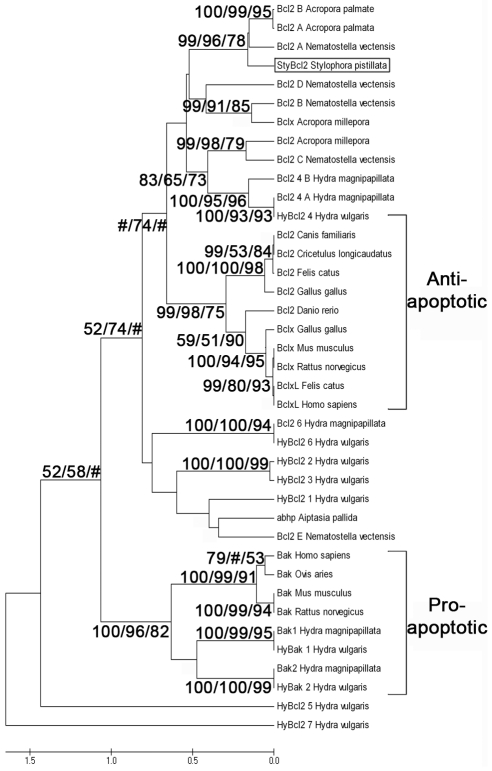
Phylogenetic relationships of metazoan Bcl-2 family members including *StyBcl*-2-like based on amino acid sequences. The alignments and UPGMA tree were obtained using the CLUSTAL-W and PAUP software. Bootstrap values (%) from 1000 bootstrap replicates are shown for neighbor-joining/maximum likelihood estimation/maximum parsimony at major nodes on the tree. Nodes receiving bootstrap values of <50% are indicated by (#). The scale bar represents genetic distance marker (amino acid substitutions). Accession numbers of sequences downloaded from GenBank are listed in Materials and Methods.

In order to establish that the necessary signaling components are present for execution and control of host apoptosis, genes playing key roles in this cellular process (i.e caspase and Bcl-2) were cloned from *S. pistillata*. For both genes, the full length cDNA was cloned and sequence analyses confirmed the identity of the transcript. The Bcl-2 transcript (*StyBcl*-2-like, accession number EU715319) encompasses 1290 bp, containing an open reading frame of 633 bp (Fig. S2). The predicted 211 amino acid sequence shares 56% similarity with the anti-apoptotic Bcl-2 and Bcl-x from vertebrates, and includes structural characteristics typifying the Bcl-2 family: Bcl-2 homology (BH) domains (i.e. BH4, BH3, BH1, BH2) and a trans-membrane domain. Phylogenetic analysis grouped *StyBcl*-2-like in a distinct cluster with vertebrate anti-apoptotic Bcl members, and with most anti-apoptotic *Hydra vulgaris* Bcl-2 members (Fig. 4).

**Figure 5:**
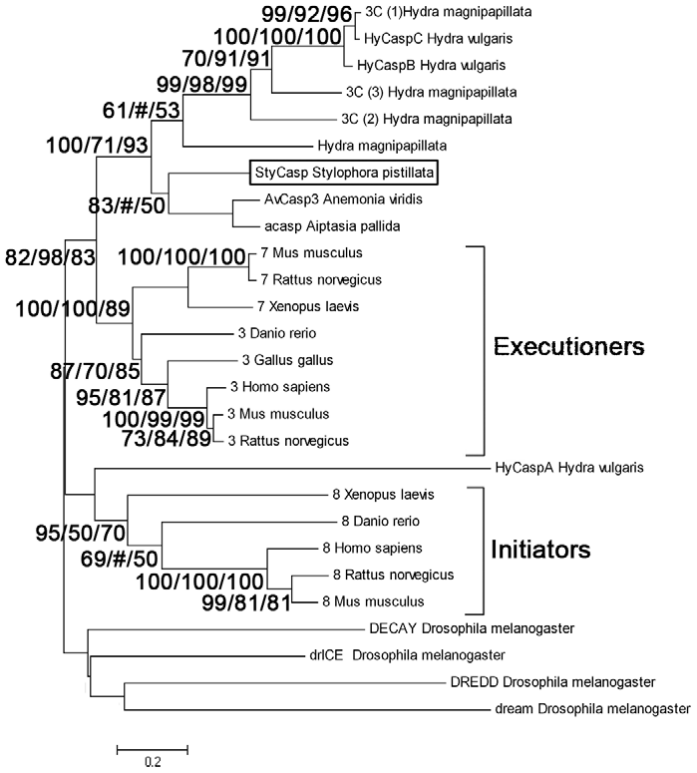
Phylogenetic relationships of metazoan caspases 3,7 and 8 including *StyCasp* based on amino acid sequences. The alignments and neighbor-joining tree sequences (large and small subunits only, excluding the prodomain) were obtained using the CLUSTAL-W and PAUP software. Bootstrap values (%) from 1000 bootstrap replicates are shown for neighbor-joining/maximum likelihood estimation/maximum parsimony at major nodes on the tree. Nodes receiving bootstrap values of <50% are indicated by (#). The scale bar represents genetic distance marker (amino acid substitutions). Accession numbers of sequences downloaded from GenBank are listed in Materials and Methods.

The caspase transcript (*StyCasp*, accession number EU715318) encompasses 1384 bp, containing an open reading frame of 1179 bp (Fig. S3). The predicted 393 amino acid sequence shares 46% and 42% similarity with executioner caspases 7 and 3 from vertebrates, respectively, and 84% similarity with *Aiptasia pallida* caspase (*acasp*). The translated *StyCasp* sequence includes a prodomain with caspase recruitment domain (CARD) resembling that found in initiator caspases, and large (p20) and small (p10) subunits resembling those found in executioner caspases. The large subunit includes highly conserved: (i) amino acids, i.e. Arg(179), His(236) and Gly(237), which are critical to substrate binding and catalysis, and (ii) caspase family cysteine active site QACXG (QACQG). The linker region contains two potential Asp tetra-peptide maturation cleavage sites (i.e. DGMD and DVTD). The small subunit contains highly conserved SWRN and GSWFI motifs, both typifying executioner caspase 3s, and are known to play an important role in substrate binding. Phylogenetic analysis consisting of only large and small subunit sequences grouped *StyCasp* and most cnidarian caspase sequences in a distinct cluster, more related to executioner vertebrate caspases 3 and 7 than to initiator caspase 8 or to executioner caspases from the invertebrate model *Drosophila melanogaster* (Fig. 5). In addition, the *StyCasp* grouped distinctly with two caspase variants (HyCasB and C) derived from *H. vulgaris*, but not with HyCaspA.

### Relative expression of StyBcl-2-like and StyCasp

The profiles of the *StyBcl*-2-like mRNA were similar to those of caspase activity throughout the experiment, also showing increased levels over time in the thermal-stressed groups, attaining maximal levels within 6–24 h of thermal stress and then declining to basal levels within 72 h (Fig. 2B). Similarly, at 34°C, the relative expression of *StyCasp* in the thermal-stressed groups increased significantly over time (6 to 48 h), reaching maximal levels within 24 h (3-fold higher compared to controls). A decline to basal levels was observed within 72 h of thermal stress (Fig. 2C, Exp. 3). However, at 32°C the relative expression of *StyCasp* increased significantly only at one time point in each experiment: at 6 h in Exp. 1 and at 24 h in Exp. 2, 2-fold and 3-fold higher compared to controls, respectively (Fig. 2C). Nonetheless, in both experiments a trend of increase could be observed between 6–24 h in the thermal-stressed groups, peaking at 24 h, and declining within 72 h.

## Discussion

Thermal stress response involves numerous aspects of the cnidarian host cellular regulation [10], [31]–[35]. A growing body of evidence indicates that apoptosis is one of the prominent host responses to thermal stress, and is dependent on the extent and duration of the stress [25], [28]–[30]. Apoptosis was also proposed to partake in coral bleaching [7]. However, only slim experimental data are available on this response at long-term thermal stress, and very few genes involved in the apoptotic cascades have been cloned so far from corals. In this study we have focused on thermal stress-induced molecular and cellular apoptotic responses in the stony coral, *S. pistillata*, from the Gulf of Eilat, under moderate (32°C) and severe (34°C) temperature regimes. These responses were monitored repeatedly over 72 to 216 h of thermal stress periods, in order to validate the consistency of the coral's responses and its ability to cope with such a stress.

Our first steps were the cloning and molecular characterization of cDNA sequences encoding for the *S. pistillata* caspase and Bcl-2 homologs. Based on the obtained sequences we were able to monitor the respective gene expression profiles and to relate them to the occurrence of apoptosis. The induction and progression of apoptosis were detected by DNA fragmentation and activation of caspase(s), the critical central proteases of this process. These results were related with the incidence of bleaching and coral survival.

As confirmed by the DNA fragmentation (TUNEL) assay, the thermal stress rapidly (at 6 to 48 h of stress) induced cell death in *S. pistillata* (Fig. 3A). However, this response appears to be restrained, as positive cells were largely restricted to the gastrodermal layer, and by 72 h of stress were hardly detected. The caspase-like activity measured biochemically in host extracts of *S. pistillata* displayed two of the caspases' typifying characteristics. First, the protease(s) cleaved the polypeptide DEVD-AFC, known to be a specific substrate of the mammalian caspase 3 [36]. Secondly, this activity was inhibited by the competitive substrate Z-VAD-FMK [37], strengthening the specific involvement of a caspase activity in host tissues of *S. pistillata*. Although contamination of host extracts with zooxanthellae proteins could not be avoided, their contribution to DEVD cleavage is allegedly minor, as demonstrated by Richier et al. in freshly isolated zooxanthellae from thermal stressed *Anemonia viridis* [25]. Hence, in our study the results of the DEVD cleavage were basically referred to as host-caspase mediated.

Based on physiological parameters (Fig. S1) the bleaching thermal threshold of *S. pistillata* was found to be 34°C. Three months following severe thermal stress, the bleached *S. pistillata* fully recovered, resembling the situation observed in natural coral populations [38]. The ability of the Red Sea *S. pistillata* to recover from the long-term thermal stress (6 weeks) and bleaching indicates that this variety is relatively resistant to thermal stress, as opposed to other varieties of this species that were studied in Sesoko Island, Japan [4], the Great Barrier Reef [39], [40] and southern Kenya [40].

Under severe thermal stress (34°C) the expression of the *StyCasp* gene increased significantly within 6 to 48 h, declined to basal levels at 72 h and maintained these levels constant up to 168 h (at which time ambient water temperature was restored) (Fig. 2C). The latter pattern paralleled with that of the caspase-3 like activity (Fig. 2A). However, under moderate thermal stress (32°C) only episodic up-regulation of the *StyCasp* was detected in the course of 6 to 24 h of stress (Fig. 2C). These results suggest that severe thermal stress stimulates both *StyCasp* activity and gene expression whereas moderate thermal stress stimulates mainly *StyCasp* activity. To attest this notion further functional studies are required. Also, we cannot rule out that the rise in caspase activity at 32°C could be the result of other caspase genes not characterized yet.

Assessment of the *StyCasp* sequence revealed characteristics typifying both initiator and executioner caspases (Fig. S3). The large prodomain includes a CARD motif found in vertebrate initiator and inflammation caspases, i.e. caspases 2, 9, 1, 5, and 4 [14], whereas the large and small subunits encompass highly conserved motifs (i.e. “QACQG”, “SWR” and “GSWFI”) and amino acids situated at conserved positions (i.e. R179 and H237) which play a key role in substrate binding, and executioner caspase 3 activation [14], [41], [42]. Such chimeric caspases were also characterized in *Hydra* [23], sponges [43] and sea anemones [24], [25]. It was hypothesized that such a CARD-containing putative executioner caspase is ancestral to the executioner caspases found in higher metazoans, which have lost the CARD domain in the course of evolution [24].

Although the function of *StyCasp* needs to be clarified, our phylogenetic analysis (Fig. 5) suggests its role as an executioner caspase. The *StyCasp* sequence was grouped distinctly with two *H. vulgaris* caspases (i.e. HyCaspB and C) as well as with *A. pallida* caspase (*acasp*). Both the *H. vulgaris* [23] and *A. pallida* [44] caspases were functionally characterized and their ability to specifically cleavage caspase 3 substrate was confirmed.

Concomitant with the caspase activity, the *StyBcl*-2-like gene expression levels significantly increased between 6 to 48 h of the thermal stress and declined to basal levels at 72 h of treatment (Fig. 2A, B). The rapid and immediate stimulation of *StyBcl*-2-like expression following the temperature treatments (Fig. 2B) could imply its importance to the host response in both moderate and severe thermal stress. The Bcl-2 family includes pro- and anti-apoptotic members, which are acting on mitochondrial and microsomal membranes [12]. This means that the up-regulation of the *StyBcl*-2 –like gene under thermal stress could be interpreted as either an anti- or pro- apoptotic response. However, our phylogenetic analyses demonstrate that *StyBcl-2*-like sequence clusters distinctly with well characterized anti-apoptotic Bcl-2 family members from *H. vulgaris* and vertebrates (Fig. 4). Moreover, within this cluster, *StyBcl*-2-like grouped specifically with HyBcl-2-like 4, which is proven to be the most potent anti-apoptotic Bcl-2 member when transiently expressed in mammalian cells [23]. Hence, an anti apoptotic role for *StyBcl*-2-like may explain the reduction of the apoptotic response in the thermal-stressed corals as the TUNEL assay results (Fig. 3) and the complete recovery of the bleached host (Fig. 1) indicated. Indeed, studies with sponges [45] and myeloid leukemic cells [46] highlighted the connection between over-expression of Bcl-2 gene under thermal stress and cell resistance to apoptosis.

Over-expression of Bcl-2 is reported to exert an anti-apoptotic effect, at least in part, by acting as a regulator of oxidative stress [12]. In this regard, thermal stress was found to accelerate ROS production in symbiotic cnidarians, mainly in the zooxanthellae partner [8]. Thus, the oxidative stress caused by ROS, either leaking from the zooxanthellae or being generated in the host cells, can initiate apoptosis in both symbiotic partners [9], [26]. Therefore, the activation of anti oxidant/anti apoptotic molecules within host cells could serve as an important mechanism diminishing oxidative stress and moderating the apoptotic response.

In various studied models activated caspases execute apoptosis giving rise to cell death [19], [20], [21], whereas Bcl-2 acts as an anti apoptotic protein, and therefore serves as a critical checkpoint for cell survival or death [17], [18]. However, the observed parallelism between caspase activity and *StyBcl*-2-like expression levels in thermal stressed corals appears to reflect a contrasting situation. Most recently, a similar parallelism was observed in *Acropora millepora* subjected to moderate thermal stress [30]. These similarities between *S. pistillata* and *A. millepora* response to thermal stress also included (i) a decline of caspase activity and Bcl-2 gene expression to basal levels within 72 h of stress, and (ii) survival and recovery of the thermal stressed corals. In an attempt to explain the contradiction between the claimed anti apoptotic nature of the *A. millepora* Bcl-2-like gene and its over-expression coinciding with the occurrence of apoptosis, Pernice et al. [30] suggested a model where thermal stress simultaneously activates (i) caspase-dependent apoptosis in the cells that have been impaired irreversibly and (ii) initiation of a coincident antioxidant response including a relative over-expression of Bcl-2 that could have a delayed mitigating effect on apoptotic activity in the surviving cell population. In a similar study carried out with the sea anemone *A. viridis*, Richier et al. [25] demonstrated thermal stress induced caspase-like activity and antioxidant capacity in the gastrodermal cells. Of note are the highly similar temporal profiles that were induced by the thermal stress in both *A. viridis* (caspase activity and antioxidant capacity) and *S. pistillata* (caspase activity and *StyBcl*-2-like transcript levels), showing elevation between 6 to 48 h, and restoration of basal levels up to 168 h of stress. Taken together, the consistent results in our 3 year study with *S. pistillata* and those obtained with *A. millepora* [30] and *A. viridis* [25] would suggest a common mechanism enabling the acclimation of Anthozoa species to thermal stress.

Although the bleaching threshold of *S. pistillata* from the Gulf of Eilat was set in this study at 34°C, caspase activity and *StyBcl*-2-like transcript levels were elevated in all experiments, even at moderate thermal stress (32°C, Fig. 2A, B). This is consistent with the work of Ainsworth et al. [27], who reported that sub-cellular and cellular responses in the host, including apoptosis, may occur at lower temperatures, before the onset of symbiont loss. In their study with *Acropora aspera*, a species susceptible to thermal stress, apoptosis of host cells was observed first in the gastrodermal cell layer and eventually progressed to the epithelial tissue layer, followed by bleaching and death of the coral. Likewise, apoptotic processes leading to bleaching and coral mortality were also documented in *S. pistillata* originated from Hawaii [31]. In this latter work caspase activity was consistently elevated up to 168 h of moderate thermal stress (32°C), at which point the bleached coral died. Exposure to caspase inhibitor has “rescued” the thermal stressed coral from death and only a moderate bleaching has occurred. Nevertheless, our data obtained with *S. pistillata* from the Gulf of Eilat clearly demonstrate that apoptosis was limited to the gastroderm, and did not progress to the epithelial tissue layer (Fig. 3A). The apoptotic markers, including DNA fragmentation and caspase profiles indicate that the apoptotic response was blocked despite the continuation of the high temperature regime. It would appear that the acute response to temperature increase was followed by acclimatization to the sustained high temperature. A similar subsequent acclimatization could also be observed phenotypically: under acute thermal stress *S. pistillata* displayed contracted polyps (Fig. 1B) that reverted to normal reopen polyps once bleaching has occurred (Fig. 1C). Hence, the symbiosis breakdown seems to be a crucial step enabling the host to acclimate to thermal stress. This could be related to symbiont susceptibility to temperature increase, as reported by Tchernov et al. [47]. The reestablishment of symbiotic associations, which occurred only when the thermal stress was discontinued, further attests this notion. However, the differential apoptotic responses to thermal stress among geographical variants of the same species, as they were documented in *S. pistillata*, could also involve an adaptation process whereby the population becomes better suited to its habitat, as suggested by Tchernov et al. [31]. Correspondingly, in their recent study employing the microarray technology and *in silico* analyses of sets of differentially expressed gene clusters, Polato et al. [48] demonstrated variation in gene expression profiles of coral embryos in response to thermal stress at two locations within a species' range. Nevertheless, further work is needed to establish the heritability of the observed changes and evaluate the ecological and evolutionary significance that this variation may have on the adaptive potential of corals in the face of environmental change.

In view of the above, a “two- stage” response in coral resistant to thermal stress can be suggested. The first stage involves the onset of apoptosis in the gastrodermal layer containing the zooxanthellae. Considering their notable activity as generators of oxidative stress [8], the symbionts' sensitivity to thermal stress appears to largely affect the activation of apoptosis in neighboring host cells. Concomitantly, cascade(s) of anti-oxidant/anti-apoptotic mediators, such as Bcl-2, are being up-regulated as “first aid” to block the progression of apoptosis to other cells. The second stage involves the acclimatization of the coral to the chronic thermal stress alongside the completion of symbiosis breakdown. As suggested by Baker [49], this process provides the corals with an option to rid themselves rapidly of suboptimal and sometimes also harmful symbionts. Yet, symbiosis breakdown seems to be efficient in “rescuing” the host only when apoptotic responses are actively moderated as part of the first-stage response. Otherwise, despite the symbiosis breakdown the bleached corals will fail to undergo acclimatization and die. Hence, the ability to rapidly curb apoptosis appears to be the most important trait attributing to coral's thermotolerance and survival.

Our results reinforce the growing body of evidence indicating that apoptosis is one of the initial host responses to thermal stress, and that cnidarians can initiate and regulate apoptosis. However, the accurate mechanisms underlying differential apoptotic responses to similar stimuli remain elusive. The challenge for future research will therefore be to functionally characterize pro- and anti-apoptotic gene networks, discover the respective sites of expression of these genes, and decipher the fundamental protective mechanism that decreases cellular sensitivity to thermal stress damaging events, so as to allow cells to escape the deadly engagement of apoptosis.

## Materials and Methods

### Collection and maintenance of corals

*S. pistillata* specimens were collected at a depth of 10 meters in the Gulf of Eilat (Red Sea, Israel). In each experiment, a single colony was targeted in order to eliminate sources of variation from coral and zooxanthellae genotypes and thermal/light history. To validate the replicability of the results, experiments were conducted during the summer time of different years and months (Exp. 1: Aug. 2008; Exp. 2: Sep. 2009; Exp. 3: June 2010). All experiments were conducted in aquaria with running ambient seawater (24±0.9°C) and exposed to shaded ambient light [10]. Aquaria were placed in large fiberglass ponds with continuous water flow to buffer temperature fluctuations; heaters were used to raise the temperature in the heated aquaria. HOBO Pendant Temperature/Light Data Loggers (Onset Corp UA-002-64) recorded temperature and light intensity every 5 min. Coral nubbins (∼2 cm long) were cut and glued onto pin-like bases. After an acclimation period of 2 weeks in one 50 L aquarium, fragments were divided evenly between two identical 20 L aquaria and acclimated again for 3 days. Time zero was sampled and the experimental aquarium was heated to 32°C [10] (Exp. 1 and 2) or to 34°C for 24 h and back to 32°C (Exp. 3). The average temperature of the experimental aquarium during the experiments was 32.09±0.3°C or 34.1±0.09°C. All experiments started at the same time of day and the same experimental setup and sampling scheme was used. The number of replicates (n) and sampling times (h) were: Exp. 1: n = 4, h = 0, 2, 6, 8, 24, 72; Exp. 2: n = 6, h = 0, 6, 12, 24, 72, 168, 216; Exp. 3: n = 6, h = 0, 6, 12, 24, 48, 72, 168. Coral fragments were snap-frozen with liquid nitrogen, and stored at −80°C. Using control for each time point eliminated differences due to time of sampling. Also, when full bleaching became evident after 7 days of thermal stress (Exp. 3), 6 fragments were transferred back to ambient temperature. The remaining fragments were kept at 32°C for one month in the experimental aquarium, and then ambient temperature was restored. Recovery of corals from bleaching was assessed using color chart (http://www.coralwatch.org).

### RNA extraction

Total RNA from all frozen coral fragments was extracted using Trizol RNA isolation reagent (Invitrogen, Carlsbad, CA) according to manufacturer's instructions with slight modifications: coral fragments were placed directly into Trizol and kept on ice for 2 h. Following isopropanol precipitation, the RNA pellet was washed three times in 80% ethanol. RNA pellets were redissolved in nuclease-free water and RNA quantity and quality were assessed with an RNA:DNA Calculator (Gene Quant pro).

RNA integrity was analyzed using 1.5% (w/v) agarose gel electrophoresis. Clear 28S and 18S rRNA bands, and an increased intensity (approximately 2-fold higher) of the 28S rRNA band compared to 18S rRNA band, were used as criteria for an intact RNA (http://www.ambion.com/techlib/tn/83/8313.html).

### RT-PCR

All primers are listed in Table S1. Total RNA from *Stylophora pistillata* was reverse transcribed using random nanomer primer (Sigma-Aldrich, MO, USA) and Bio-RT (Bio-Lab, Jerusalem, Israel). Degenerate primers for *StyCas*p were designed from two highly conserved regions of caspase 3 amino acid sequences from phylogenetically distant organisms (*Hydra vulgaris* AAF98012, *Homo sapiens* AAH15799, *Aiptasia pallida* DQ218058 and *Anemonia viridis* DQ097195): CasF1 and CasR3a. For *StyBcl*-2-like, degenerate primers were designed from amino acid sequences of the BH1 and BH2 domains from phylogenetically distant organisms [24]: Bcl-F2 and Bcl-R2. cDNA was PCR amplified using 300 nM of each primer and Fast Start High Fidelity PCR system (Roche, IN, USA). PCR products were purified using PCR purification kit (QIAGEN, Hilden, Germany), ligated in to the pGEM-T easy vector (Promega, Madison, USA), cloned and sequenced (Hy-Labs, Rehovot, Israel).

### Rapid amplification of cDNA ends (RACE)

To further obtain the full-length cDNA sequence of *S. pistillata* caspase and Bcl, 5′/3′ SMART RACE cDNA Amplification Kit and Advantage® 2 Polymerase (both from Clontech, CA, USA) were used according to manufacturer instructions. All primers are listed in Table S1. For 3′-RACE, the gene specific primers were: StyCasF1, StyCasF2 for *StyCas*p and StyBcl F3, StyBcl F4 for *StyBcl*-2-like. For 5′-RACE, the gene specific primers were: StyCasR1, StyCasR2 for *StyCas*p and StyBcl-2 R3, StyBcl-2 R4 for *StyBcl*-2-like. To confirm the generated sequence of the full open reading frame primers were: StyCasF4 and StyCasR4 for *StyCas*p, StyBcl-2 F5 and StyBcl-2 R5 for *StyBcl*-2-like. PCR products were analyzed, ligated, cloned and sequence as described above.

### Quantitative real-time PCR (qRT–PCR)

qRT–PCR primers (Table S1) were designed using primer express 3.0 (Applied Biosystems). Total RNA (700 ng) was treated with RQ1 Dnase (Promega, WI, USA) and reverse transcribed using High Capacity cDNA kit (Applied Biosystems, CA, USA). Transcript levels were determined by realtime PCR using the 7500 Fast Real-Time PCR system (Applied Biosystems, CA, USA). Triplicate first strand cDNA aliquots (2 µl) from each sample served as templates for PCR using Power SYBR Green PCR Master Mix (Applied Biosystems, CA, USA) and 300 nM genespecific primers. Amplification reactions were carried out following the manufacturer's instructions. The copy number of *StyBcl*-2-like and *StyCasp* in unknown samples was determined by comparing CT values with two internal control genes, 18s rRNA and adenosyl-homocysteinase (AdoHcyase) from *S. pistillata* host tissue.

### Tissue extractions, zooxanthellae counts and surface area

All steps were conducted on ice. Tissue (n = 6) was removed of the skeleton by a toothbrush into 1–2 ml of phosphate buffered saline (PBS, pH = 7.6). The extract was homogenized with Dounce homogenizer and centrifuged at 4000 rpm, 4°C for 10 min to pellet intact zooxanthellae cells. Supernatant was collected, homogenized again and centrifuged at 14,000 rpm, 4°C for 20 min. Supernatant was collected, its volume recorded, and total protein concentration of each sample was measured using the Bradford protein assay and a BSA protein standard (both from BioRad, CA, USA) according to the Bradford method [50]. Supernatant was divided into aliquots and stored at −80°C for further analysis. The pellet containing zooxanthellae was processed according to standard methods [51]. Estimates of the surface area of individual *S. pistillata* fragments were obtained according to Stimson and Kinzie [52]. Bleaching was determined according to Glynn and D'cros [53].

### Caspase assay

Caspase 3-like activity was assayed fluorometrically using the specific substrate Z-DEVD-AFC (Z-Asp-Glu-Val-Asp-AFC) (Calbiochem, Darmstadt, Germany) as described [54]. For inhibition experiments, Z-VAD-FMK (Z-Val-Ala-Asp-CH2F*) (CalBiochem, Darmstadt, Germany) was used as described [31]. Caspase activity was expressed as RFU/µg protein^−1^ h^−1^.

### Sample preparation and in situ detection of cell death

Coral fragments were fixed individually in 4% paraformaldehyde in 3× phosphate buffered saline (PBS, pH 8.2) for 12 h at 4°C, decalcified using 20% (w/v) EDTA in 3× PBS (pH 8.2) and then stored in PBS at 4°C. Tissues were dehydrated in ethanol series, cleared in n-butyl alcohol and then embedded into paraffine. Lengthwise sections of 7 µm thick were attached to Superfrost Plus slides (Menzel, Brauschweig, Germany). Apoptotic cells were detected *in situ* using terminal deoxynucleotidyl transferase (TdT) biotin-dUTP nick end labeling (TUNEL) technique (DeadEnd_TM_ colourimetric detection kit, Promega, Madison, WI, USA) as per manufacturers recommendations. As a positive control, slides were treated with DNase I, and as a negative control, slides were treated with the TUNEL mix without the rTdT enzyme as suggested by the manufacturer. Slides were counterstaining with 0.3% methyl green (Sigma-Aldrich, St. Louis, MO, USA). Cells were defined as apoptotic if positively labeled as indicated by brown stain as opposed to the green counterstained non-apoptotic cells. Cell death quantification was determined by counting cells using the UTHSCSA Image Tool program (maxrad6.uthscsa.edu). TUNEL-labeled nuclei were counted in coral sections obtained from 3 individual coral fragments (5 sections were analysed and averaged per coral fragment), using the same magnification (x40) and compared to the total number of host cells in the same image field to obtain a percentage of TUNEL-positive host cells.

### Downloaded Sequences

#### Sequences downloaded from GenBank for Fig. 4

Vertebrates: zBak *H. sapiens* (4502363), Bak *Ovis aries* (9621788), Bak *Mus musculus* (6671612), Bak *Rattus norvegicus* (8050832). Bcl2 *Canis familiaris* (40846417), Bcl2 *Cricetulus longicaudatus* (15213854), Bcl2 *Felis catus* (25166611), Bcl2 *Gallus gallus* (231635), Bcl2 *Danio rerio* (18858349), Bclx *G. gallus* (539495), Bclx *R. norvegicus* (1083601), Bclx *M. musculus* (2493277), Bclxl *F. catus* (19570354), Bclxl *H. sapiens* (2118487). *Hydra magnipapillata*: Bcl2 6 (221118699), Bcl2 4 (A) (221091026), Bcl2 4 (B) (221119340), Bak1 (37511437), Bak2 (52859456). *H. Vulgaris:* HyBcl-2-like 1 (ABL01493), HyBcl-2-like 2 (ABL01494), HyBcl-2-like 3 (ABS84174), HyBcl-2-like 4 (ABS84173), HyBcl-2-like 5 (ABS84172), HyBcl-2-like 6 (ABS84171), HyBcl-2-like 7 (ABS84170), HyBak-like 1 (ABL01492), HyBak-like 2 (ABS84169). *Nematostella vectensis*: Bcl2 A (156362497), Bcl2 B (156373857), Bcl2 C (156388832), Bcl2 D (208969), Bcl2 E (246637). Others: *Acropor*a *millepora*: Bcl2 (161334687), Bclx (222806072). *Acropora palmate*: Bcl2 B (169231861), Bcl2 A (71778899). *A. pallid*: *abhp* (ABA61360).

#### Sequences downloaded from GenBank for Fig. 5

Caspase 3s: *D. rerio* (BAB32409), *G. gallus* (NP_990056), *H. sapiens* (1651687), *M. musculus* (6753284), *R. norvegicus* (6978605). Caspase 7s: *R. norvegicus* (1560075), *M. musculus* (6680850), *Xenopus laevis* (7619904). Caspase 8s: *D. rerio* (52138561), *H. sapiens* (15718708), *M. musculus* (4138211), *R. norvegicus* (11560103), *X. laevis* (76199060). Caspases from *H. magnipapillata*: (221104324), 3c (3) (XP_002165630), 3c (2) (XP_002165630), 3c (1) (XP_002165572). Caspases from *H. vulgaris*: HyCaspA (AAF98011), HyCaspB (AAF98012), HyCaspC (AAX18880). Caspases from *Drosophila melanogaster*: DECAY (17137722), DREDD (24638925), drICE (1929040), STRICA (19921676). Others: *A. pallid*: *acasp* (77158000). *A. viridis*: AvCasp3 (74040399).

### Sequence and statistical analysis

Sequence alignments were performed with CLUSTAL-W (EMBLEBI) embedded in BIOEDIT (version 5.0.9) software [55] and phylogenetic trees were constructed using the PAUP software [56]. Statistical significance of caspase activity, *StyBcl*-2-like and *StyCasp* transcription levels between the control and thermal stressed group at each sampling point was tested using One-Way ANOVA and a t-test. Statistical significance of the mean percentage of TUNEL positive cells between groups was determined using a One-Way Anova with Tukey post-hoc testing. Analysis was undertaken with JMP (ver. 7.0.1) statistical software (SAS Institute Inc., Cary, NC).

## Supporting Information

Fig. S1 **Areal zooxanthellae density protein content.** (A) Areal zooxanthellae density and (B) areal protein content in corals incubated in control (white) or subjected to thermal stress of 168 h (gray). Fragments of *S. pistillata* were placed in two aquaria (control at 24°C and thermal stress at 34°C for 24 h and then back to 32°C (Exp. 3)). Results are expressed as means ± SE of independent extractions from 6 distinct fragments.
(TIF)

Fig. S2 **Nucleotide and deduced amino acid sequence of** ***S. pistillata*** **Bcl-2 (** ***StyBcl*** **-2-like) cDNA.** Boxed residues indicate Bcl-2 homology domains (BH domains) BH4, BH3, BH1, BH2 and the Trans Membrane (TM) domain.
(TIF)

Fig. S3 **Nucleotide and deduced amino acid sequence of** ***S. pistillata*** **caspase (** ***StyCasp*** **) cDNA.** Putative prodomain sequence appears in italic characters, the large (p20) subunit in bold italic characters, linker region in regular type and the small subunit in bold regular type. Asterisks indicate amino acids essential to substrate binding and catalysis. Residues boxed are: caspase family cysteine active site QACQG (box I), linker region with two potential Asp tetrapeptide maturation cleavage sites DGMD or DVTD (box II) and highly conserved motifs important in substrate binding and typical of executioner caspase 3s, SWRN and GSWFI (box III and IV, respectively).
(TIF)

Table S1 Primers utilized in the study. qPCR: Primers for real time PCR; F: Forward; R: Reverse.
(DOCX)
