# Supplementary figures and images for: Regulation of Apoptotic Pathways by Stylophora pistillata (Anthozoa, Pocilloporidae) to Survive Thermal Stress and Bleaching
Source: PLoS One. 2011 Dec 14;6(12):e28665. doi: 10.1371/journal.pone.0028665 (PMC3237478; doi:10.1371/journal.pone.0028665)

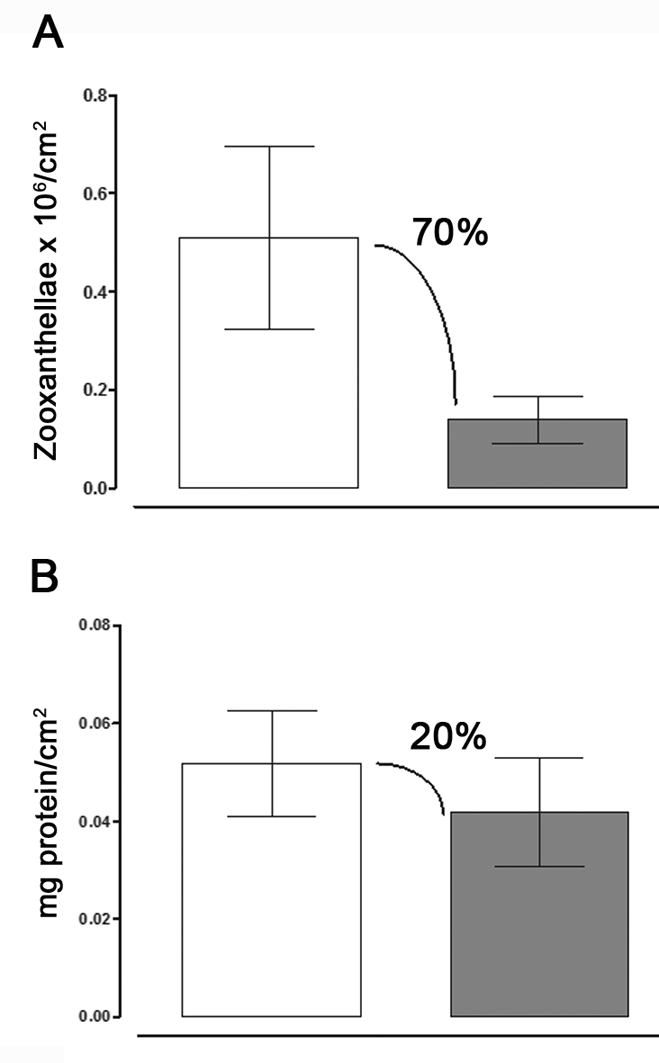

Supplement: Fig. S1 — Areal zooxanthellae density protein content. (A) Areal zooxanthellae density and (B) areal protein content in corals incubated in control (white) or subjected to thermal stress of 168 h (gray). Fragments of S. pistillata were placed in two aquaria (control at 24°C and thermal stress at 34°C for 24 h and then back to 32°C (Exp. 3)). Results are expressed as means ± SE of independent extractions from 6 distinct fragments. (TIF) [file pone.0028665.s001.tif]

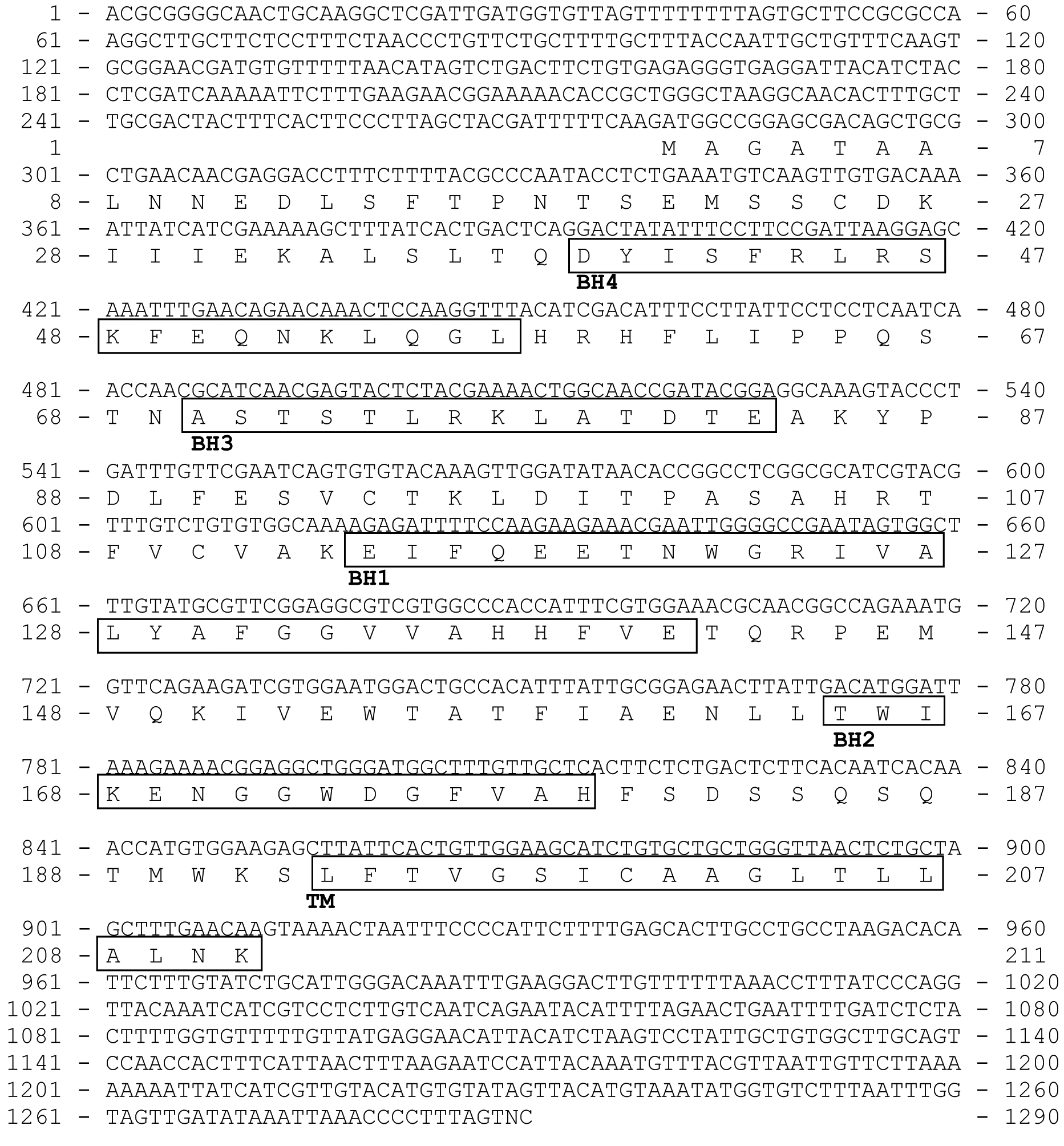

Supplement: Fig. S2 — Nucleotide and deduced amino acid sequence of S. pistillata Bcl-2 ( StyBcl -2-like) cDNA. Boxed residues indicate Bcl-2 homology domains (BH domains) BH4, BH3, BH1, BH2 and the Trans Membrane (TM) domain. (TIF) [file pone.0028665.s002.tif]

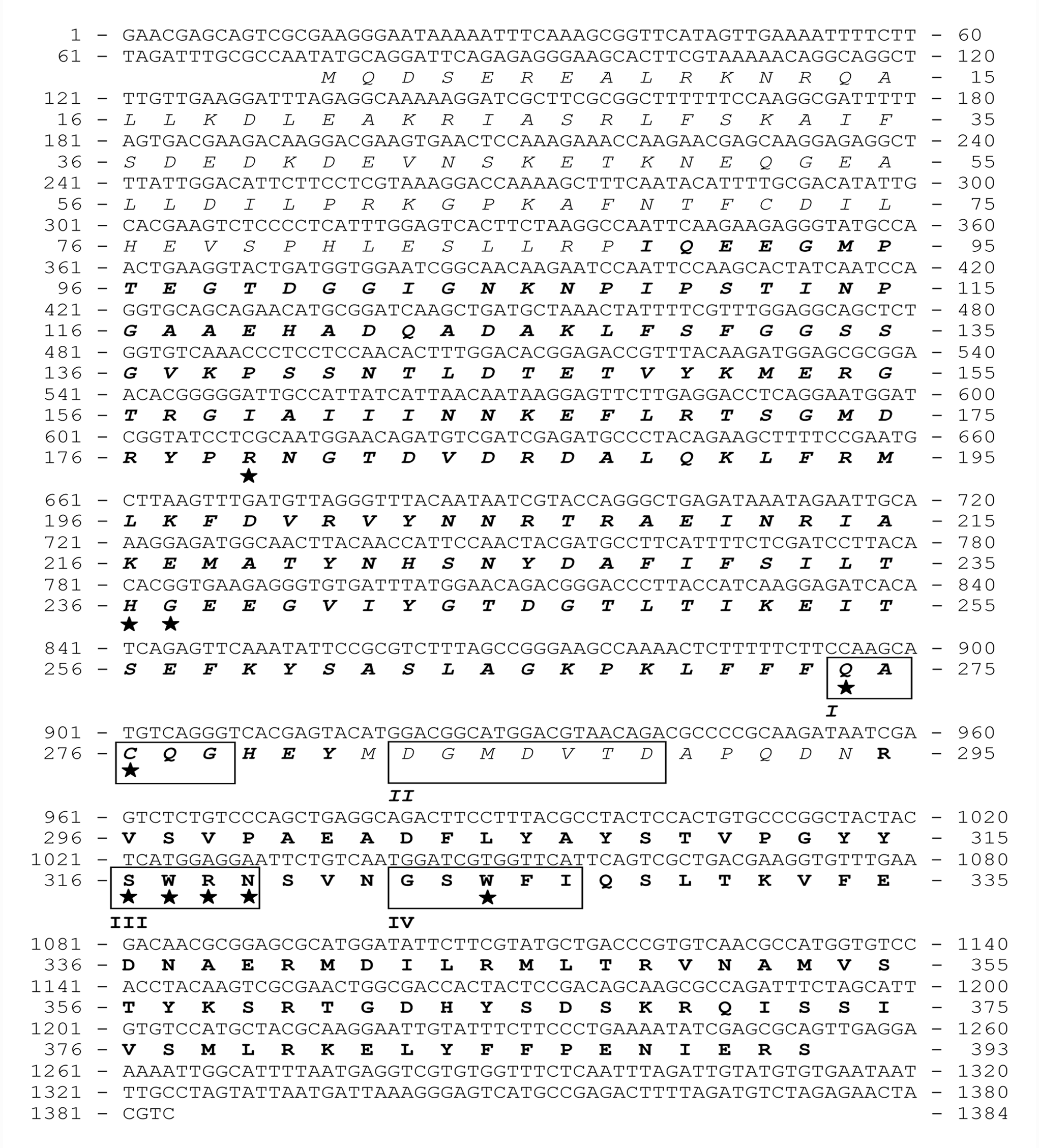

Supplement: Fig. S3 — Nucleotide and deduced amino acid sequence of S. pistillata caspase ( StyCasp ) cDNA. Putative prodomain sequence appears in italic characters, the large (p20) subunit in bold italic characters, linker region in regular type and the small subunit in bold regular type. Asterisks indicate amino acids essential to substrate binding and catalysis. Residues boxed are: caspase family cysteine active site QACQG (box I), linker region with two potential Asp tetrapeptide maturation cleavage sites DGMD or DVTD (box II) and highly conserved motifs important in substrate binding and typical of executioner caspase 3s, SWRN and GSWFI (box III and IV, respectively). (TIF) [file pone.0028665.s003.tif]
