# Supplementary material for: Regulation of Apoptotic Pathways by Stylophora pistillata (Anthozoa, Pocilloporidae) to Survive Thermal Stress and Bleaching
Source: PLoS One. 2011 Dec 14;6(12):e28665. doi: 10.1371/journal.pone.0028665 (PMC3237478; doi:10.1371/journal.pone.0028665)
Supplement: Table S1 — Primers utilized in the study. qPCR: Primers for real time PCR; F: Forward; R: Reverse. (DOCX) [file pone.0028665.s004.docx]

| Primer name | Sequence 5`-3` |
| --- | --- |
| Cas F1 | CAYGGNGARGARGG |
| Cas R3a | CCANGARTARTANCC |
| Bcl F2 | AAYTGGGGNCGNGTNGT |
| Bcl R2 | CCANCCNCCRTTYTTNCKDATCCA |
| StyCas F1 | GGAACAGACGGGACCCTTACCATC |
| StyCas F2 | TCAGGGTCACGAGTACATGGACGGC |
| StyBcl F3 | CGGAGGCGTCGTGGCCCACCATTTCG |
| StyBcl F4 | CGCAACGGCCAGAAATGGTTCAG |
| StyCas R1 | GGGCACAGTGGAGTAGGCGTA |
| StyBcl-2 R3 | CTGAACCATTTCTGGCCGTTGCG |
| StyBcl-2 R4 | GAAATGGTGGGCCACGACGCCTCCG |
| StyCas F4 | CGAGCAGTCGCGAAGGGAATA |
| StyCas R4 | GACGTAGTTCTCTAGACATCTAAAAGTC |
| StyBcl-2 F5 | ACGCGGGGCAACTGCAAGGCTC |
| StyBcl-2 R5 | TTCAGTTCTAAAATGTATTCTGATTGAC |
| qPCR StyCas F2 | GGACGGCATGGACGTAACAG |
| qPCR StyCas R2 | CAGCTGGGACAGAGACTCGAT |
| qPCR StyBcl F2 | CGTCGTGGCCCACCATT |
| qPCR StyBcl R2 | CCACGATCTTCTGAACCATTTCT |
| qPCR 18s Coral F | AACGATGCCAACTAGGGATCA |
| qPCR 18s Coral R | GGTTTCCCATAAGGTGCCAAA |
| qPCR Adenozyl F2 | CAGGCTGTCTGCACATGACAA |
| qPCR Adenozyl R2 | TCCCAATTCAGTCAATGTTTCAA |
